# Hatching and Mortality of *Meloidogyne enterolobii* Under the Interference of Entomopathogenic Nematodes In vitro

**DOI:** 10.21307/jofnem-2019-058

**Published:** 2019-09-17

**Authors:** Alixelhe Pacheco Damascena, Júlio César Antunes Ferreira, Marylia Gabriella Silva Costa, Luis Moreira de Araujo Junior, Silvia Renata Siciliano Wilcken

**Affiliations:** 1 Universidade Estadual Paulista (UNESP), Faculty of Agronomic Sciences, Department of Plant Protection, 18610-034, Botucatu, São Paulo, Brazil; 2 University Federal of Espírito Santo - UFES, Department of Agronomy, Laboratory of Entomology/NUDEMAFI, Alegre, ES, Brazil

**Keywords:** Biological control, Entomopathogens, Management, Phytopathogenic nematodes

## Abstract

Plant parasitic nematodes have become one of the main problems in the tomato cultivation. Among these, *Meloidogyne enterolobii* presents great challenges to the farmer, since it is a polyphagous species and difficult to control. The entomopathogenic nematodes (EPNs) present as potential for biological control of this pathogen. The objective of the study was to evaluate the interference of EPNs *S. brazilense*, *S. feltiae*, *S. rarum*, *H. amazonensis* and *H. bacteriophora* on hatching and mortality of *M. enterolobii*. 500 eggs of this nematode and 1.000 infective juveniles of each EPN species were placed in a plastic pot totaling 25 mL of suspension and kept in an incubator at 25°C. The number of juveniles hatched in the suspension was counted every 2 days, until 10 days. After 10 days of evaluations, the remaining suspension (15 mL) containing *M. enterolobii* and EPNs was inoculated into Rutgers tomato seedlings. The suspension contained approximately in 300 eggs of *M. enterolobii* occasional juveniles and 600 IJ of each nematode species. Sixty days after inoculation were evaluated gall indexes, egg mass indexes, total number of eggs and juveniles of *M. enterolobii* and reproductive factor was calculated. In the mortality experiment, 500 infective juveniles of *M. enterolobii* and 1.000 juveniles of each EPN species were placed in a plastic pot totaling 25 mL of suspension. The evaluation of juvenile mortality was performed by counting of the mobile and immotile nematodes, by adding two drops of NaOH to the nematode suspension. It was verified that on the 10th day all ENPs provided reduction in the hatching of *M. enterolobii*. In the pot experiment it was found thato gall index, egg mass indexm, nematodes total number and reproduction factor were significantly reduced in treatments with all species of EPNs tested. However, in the mortality test, only EPNs *S. brazilense* and *S. rarum* provided mortality on the second day and *H. bacteriophora* affected mortality on the 4th day. In the other evaluations, there was no statistical difference. The results highlight the potential of the use of EPNs in programs of integrated management of *M. enterolobii* in tomato.

The tomato (*Solanum lycopersicum* L.) is one of the most cultivated oleraceous in Brazil, with a total area of 54,051 hectares and a production of 3,472.55 tons, with the states of São Paulo and Goiás being the largest producers (IBGE, 2016). In view of the socioeconomic importance of the crop and the need for controlled environmental conditions for cultivation, the tomato plants were grown in a protected environment, allowing an increase in production. However, it favored the development of several phytosanitary problems, among them, phytopathogenic nematodes (Rosa et al., 2015).

Plant parasitic nematodes have become one of the main problems in the olericulture cultivation in general. Among these, the *Meloidogyne* spp. genus representes the group of greater importance (Silva et al., 2014). The species of this genus can cause 100% losses in the production and therefore are considered one of the most harmful in the olericulture cultivation (Kurozawa and Pavan, 2005; Perry and Moens, 2006).

*Meloidogyne enterolobii* (Rhabditida: Meloidogynidae) has been shown to be a major challenge to producers (Yang and Eisenback, 1983), because it is considered polyphagous and it has a high degree of dissemination and multiplication (Carneiro et al., 2006). The *M. enterolobii* species was described from parasitism on roots of *Enterolobium contortisiliquum* (Vell.) (Fabales: Fabaceae) in China. *Meloidogyne enterolobii* has a wide range of hosts, high breeding rate, high aggressiveness and ability to overcome existing sources of resistance to other species of *Meloidogyne* (Brito et al., 2007; Cantu et al., 2009).

Several methods are studied for the control of phytonematodes (Rosa et al., 2015; Silva et al., 2014). Studies show that entomopathogenic nematodes (EPNs) have potential for biological control of these parasites (Pérez and Lewis, 2004; Lewis and Grewal, 2005).

In view of the above, the objective of the present study was to evaluate the hatching, mortality and infectivity of hatched juvenis of *M. enterolobii* to tomato in the presence of *Steinernema brazilense* PONTO2C, *S. feltiae* (Filipjev) IBCB47, *S. rarum* (Doucet) PAM25 (Rhabditida: Steinernematidae), *Heterorhabditis amazonensis* IBCB10 and *H. bacteriophora* (Poinar) HB (Rhabditida: Heterorhabditidae) in vitro, in order to verify if the EPNs promote mortality or interfere in the hatching of juveniles of *M. enterolobii.*

## Material and methods

The experiments were carried out in incubator chambers (biochemical oxygen demand—BOD) at 25°C, 70% of relative humidity, of the Laboratory of Agricultural Nematology of the Department of Plant Protection of the College of Agronomic Sciences at the Paulista State University “Júlio de Mesquita Filho”, Botucatu-SP.

The pure population of *M. enterolobii* used in the experiments was obtained from tomato plants kept in greenhouse as an inoculum source and multiplied in ‘Rutgers’ tomato in 1,000 cm^3^ pots, containing substrate composed of soil, sand and organic matter in the proportion of 1: 2: 1 (v: v: v), pre-sterilized by autoclaving (120°C for 2hr). The nematode eggs were extracted from tomato roots by the method of Hussey and Barker (1973) and concentration of the suspension determined with the aid of the Peters chamber under a light microscope.

The populations of EPNs were obtained from the EPNs Collection of the Bank of the Biological Institute of São Paulo, São Paulo, Brazil. The infective juveniles (IJs) of *S. brazilense*, S*. feltiae*, *S. rarum*, *H. amazonensis*, and *H. bacteriophora* were multiplied in third to fifth larval instar of *Diatraea saccharalis* (Fabr.) (Lepidoptera: Crambidae). Five larvae per petri dish (9 cm in diameter) were coated with filter paper moistened with a nematode suspension at the concentration of 500 IJ/larvae. The dead larvae of *D. saccharalis* were transferred to traps of White (White, 1927) and stored in incubator (BOD) at 25°C. The IJs were collected in a water film (1 cm depth) in Erlemeyers, which were kept in a BOD chamber at a temperature of 18°C, 70% of relative humidity and used up to 48 hr after collection.

For the tomato in pots test, the ‘Rutgers’ tomato seeds were placed for germination in polypropylene trays containing 128 cells containing substrate. After germination, tomato seedlings were transplanted into pots for 1000 cm^3^ containing substrate composed of soil, sand, and organic matter in the proportion of 1: 1: 1 (v: v: v), sterilized in an autoclave (120°C for 2 hr).

### Interference of EPNs in the hatching of Meloidogyne enterolobii

In this experiment, 500 eggs of *M. enterolobii* and 1.000 IJ of each EPN species were placed in a plastic pot (50 mL) dispersed in 25 mL of suspension and kept in an incubator (BOD) at 25°C. The number of hatched J2 in the suspension was counted every 2 days, until 10 days. For counting, 1 ml of suspension was withdrawn and the nematodes counted in Peters’s chamber under light microscope. The experiment was conducted in a completely randomized design with six treatments (T1 = *M. enterolobii;* T2 = *M. enterolobii + S.brazilense;* T3 = *M. enterolobii + S. rarum;* T4 = *M. enterolobii + S. feltiae;* T5 = *M. enterolobii + H. amazonensis;* T6 = *M. enterolobii + H.bacteriophora)*, and six replicates.

### Infectivity of eggs and J2 of Meloidogyne enterolobii treated with EPNs

After 10 days of evaluations, the remaining suspension (15 mL) containing *M. enterolobii* and EPNs was inoculated into Rutgers tomato seedlings, previously transplanted into 1.000 cm³ pots containing substrate composed of soil, sand, and organic matter in the proportion of 1: 1: 1 (v: v: v), sterilized in an autoclave (120°C for 2 hr). In each pot, was added the suspension containing approximately 300 eggs and eventual juveniles of *M. enterolobii* and 600 IJ of each EPNs species, separately.

Sixty days after inoculation, the root system of each plant was washed in running water and subjected to B floxin staining to obtain gall indexes (GI) and egg mass indexes (EMI) according to the scale of grades proposed by Taylor and Sasser (1978), where grade 0 = no galls or egg masses; grade 1 = 1 to 2 galls or egg masses; grade 2 = 3 to 10 galls or egg masses; grade 3 = 11 to 30 galls or egg masses; grade 4 = 31 to 100 galls or egg masses and grade 5 = more than 100 galls or egg masses per root.

For the extraction of the eggs, the root system was cut into small pieces (approximately 1 cm of length) processed by the method described by Hussey and Barker (1973) in which the root system is ground in blender with sodium hypochlorite solution, followed by sieving. The total number of eggs and juveniles of *M. enterolobii* per root system was quantified with the aid of a light microscope and Peters' slides. The reproductive factor was calculated using the formula RF = FP/IP, where FP = nematode final population and IP = nematode initial population (IP = 300 eggs). For greater data reliability, the experiments were conducted twice.

### Interference of EPNs in mortality of J2 of Meloidogyne enterolobii

To obtain the second stage juveniles (J2) to be used in the experiments, the suspension containing eggs of *M. enterolobii* was placed in Baermann apparatus modified to shallow containers, according to Southey (1986).

The collections of J2 were performed every 24 hr until reaching 72 hr. Thus, 500 J2 of *M. enterolobii* and 1.000 IJ of each EPN species were placed in plastic pots, totaling 25 mL of suspension. Mortality was evaluated every other day, for 10 days. The evaluation of *M. enterolobii* mortality was performed by counting the mobile and immotile nematodes according to the methodology described by Chen and Dickson (2000), which consists of adding two drops of 1 N NaOH to the nematode suspension. The counting was done soon after, considering deads the straight and immobile nematodes, and, alive, the twisted ones.

### Statistical analyses

The experiments were conducted in a completely randomized experimental design, in a split plots scheme, with six replications. The factor with the treatments was evaluated in the plots; and the days in the subplots. The data were submitted to analysis of variance, comparing the means by the Scott–Knott test at 5% probability. The analyzes were carried out in the R Development Core Team software (2009). For greater data reliability, the experiments were conducted twice.

## Results

### Interference of EPNs in the hatching of Meloidogyne enterolobii

The use of EPNs affected the hatching of *M. enterolobi*. In the first evaluations (2nd and 4th day) there was no statistical difference between treatments (*P* < 0.005). However, on the 6th day, all treatments differed statistically from the control, providing a reduction in hatching of *M. enterolobii*. On the 8th day, only the treatment composed of *S. rarum* (SR) and *H. bacteriophora* (HB) provided reduction in hatching. In the last evaluation (10th day), all EPNs provided a reduction in hatching of *M. enterolobii* (*P* < 0.005) (Table 1). The maximum number of *M. enterolobii* hatched in the control and in the treatments composed by *S. rarum* (SR), *S. feltiae* (SF) and *H. bacteriophora* (HB) was on the 10th day. For *S. brazilian* (SB) and *H. amazonensis* (HA) treatments, maximum hatching occurred on day 8, and did not differ statistically from day 10.

**Table 1.** Hatching percentage of *Meloidogyne enterolobii* under the interference of entomopathogenic nematodes.

	**1**^**st**^ **Experiment**				
**Trat**	**2° day**	**4° day**	**6° day**	**8° day**	**10° day**
	**2**^**nd**^ **Experiment**				
**Trat**	**2° day**	**4° day**	**6° day**	**8° day**	**10° day**
Control	10.8 ± 3.7 aA	14.5 ± 6.7 aA	32.9 ± 16.7 aB	31.9 ± 10.6 aB	74.8 ± 15.4 aC
SB	3.3 ± 1.4 aA	14.6 ± 6.1 aA	14.0 ± 8.2 bA	43.1 ± 16.9 aB	40.8 ± 11.3 cB
SR	5.8 ± 2.0 aA	13.8 ± 4.1 aA	24.5 ± 13.4 bB	14.6 ± 10.3 bA	53.1 ± 10.3 bC
SF	2.5 ± 1.1 aA	7.7 ± 2.4 aA	11.9 ± 5.5 bA	32.9 ± 9.8 aB	57.8 ± 17.6 bC
HA	4.2 ± 3.7 aA	13.0 ± 6.1 aA	24.5 ± 8.5 bB	40.5 ± 7.8 aC	41.4 ± 13.7 cC
HB	1.7 ± 0.9 aA	20.7 ± 8.1 aB	27.3 ± 10.2 bC	15.8 ± 7.3 bB	41.3 ± 10.2 cD
F_25_	5.16*				
Control	9.2 ± 5.8 aA	15.4 ± 12.9 aA	35.7 ± 11.4 aB	26.6 ± 10.5 aB	63.5 ± 18.3 aC
SB	4.2 ± 2.9 aA	14.6 ± 6.7 aA	14.0 ± 9.1 bA	50.7 ± 14.2 aB	53.3 ± 13.7 bB
SR	10.0 ± 4.9 aA	14.6 ± 6.8 aA	22.1 ± 15.1 bB	18.4 ± 4.0 bA	53.6 ± 22.9 bC
SF	3.3 ± 2.0 aA	18.4 ± 8.7 aA	14.0 ± 6.9 bA	20.9 ± 10.0 aB	46.8 ± 13.3 bC
HA	1.8 ± 1.0 aA	13.0 ± 7.9 aA	22.2 ± 5.1 bB	40.5 ± 6.2 aC	48.2 ± 19.9 bC
HB	3.3 ± 2.0 aA	16.9 ± 7.5 aB	16.1 ± 3.1 bB	23.3 ± 7.5 bC	46.5 ± 19.3 bC
F_25_	4.49*				

### Infectivity of eggs and J2 of Meloidogyne enterolobii treated with EPNs

It was possible to verify that the gall index (GI), egg mass index (EMI), nematodes total number (NT) and reproduction factor (RF) were significantly reduced in treatments with all species of EPNs tested (*P* < 0.005). It was verified that in all the treatments there was penetration of *M. enterolobii*, however the reproduction factor in the treatments with EPNs was reduced considerably. The viability of the inoculum was confirmed in the two experiments, with RF = 2.6 and 5.7, respectively (Table 2).

**Table 2.** Egg mass index (IMO), Galls index (IG), total number of nematodes (NT) and reproduction factor (FR) of *Meloidogyne enterolobii* in roots of tomato ‘Rutgers’ under the interference of entomopathogenic nematodes.

	**1**^**st**^ **Experiment**			
**Trat**	**IMO**^**a**^	**IG**^**a**^	**NT**^**b**^	**FR**^**c**^
	**2**^**nd**^ **Experiment**			
**Trat**	**IMO**^**a**^	**IG**^**a**^	**NT**^**b**^	**FR**^**c**^
Control	4.5 ± 0.5 a	4.5 ± 0.5 a	782.0 ± 137.7 a	2.6 ± 0.5 a
SB	2.8 ± 0.7 b	2.8 ± 0.7 b	388.2 ± 56.8 b	1.3 ± 0.2 b
SR	1.5 ± 0.5 b	1.5 ± 0.5 b	376.2 ± 50.2 b	1.3 ± 0.2 b
SF	1.8 ± 1.7 b	1.7 ± 1.5 b	450.5 ± 84.1 b	1.5 ± 0.3 b
HA	1.7 ± 1.0 b	1.2 ± 1.1 b	185.8 ± 19.9 d	0.6 ± 0.1 d
HB	2.3 ± 1.0 b	2.0 ± 1.4 b	304.7 ± 77.3 c	1.0 ± 0.3 c
F_5, 30_	7.19	7.98	38.28	4.64
Control	4.2 ± 0.7 a	4.2 ± 0.7 a	1632.5 ± 296.7 a	5.7 ± 0.5 a
SB	2.7 ± 0.5 b	2.7 ± 0.5 b	770.2 ± 41.3 c	2.6 ± 0.1 c
SR	2.5 ± 0.8 b	2.7 ± 0.8 b	659.0 ± 39.3 c	2.2 ± 0.1 d
SF	2.2 ± 0.9 b	2.3 ± 1.2 b	732.0 ± 42.3 c	2.4 ± 0.1 c
HA	2.7 ± 0.8 b	2.8 ± 1.1 b	641.7 ± 449.0 c	2.1 ± 1.4 d
HB	2.2 ± 1.1 b	2.0 ± 1.1 b	926.5 ± 21.9 b	3.1 ± 0.1 b
F_5, 30_	4.38	3.59	49.74	154.16

### Interference of EPNs in mortality of J2 of Meloidogyne enterolobii

The mortality of *M. enterolobii* in the first evaluation (day 2) was 76.2 in experiment 1 and 102.7 in experiment 2 with *S. brazilense* (SB) and *S. rarum* (SR) provided a mortality of 87.2 in the experiment 1 and 87.8 in experiment 2, differing statistically from the other treatments. On day 4, *H. bacteriophora* (HB) provided mortality of 121.0 and 154.0 *M. enterolobii* juveniles. On the 6th, 8th, and 10th day there was no statistical difference between treatments. In general, the presence of EPNs in the suspension caused mortality of the *M. enterolobii* IJ only in the first two evaluations, 2° and 4° day (Table 3).

**Table 3.** Mortality percentage of juveniles of *M. enterolobii* under the interference of entomopathogenic nematodes.

	**1**^**st**^ **Experiment**				
**Trat**	**2° day**	**4° day**	**6° day**	**8° day**	**10° day**
	**2**^**nd**^ **Experiment**				
**Trat**	**2° day**	**4° day**	**6° day**	**8° day**	**10° day**
Control	6.8 ± 2.5 aA	5.2 ± 0.7 aA	7.7 ± 3.1 aA	10.3 ± 0.6 aA	17.3 ± 6.2 aB
SB	15.2 ± 4.0 bB	11.9 ± 2.0 aA	8.9 ± 3.0 aA	5.8 ± 0.4 aA	12.5 ± 4.6 aB
SR	17.4 ± 4.8 bA	14.4 ± 1.0 aB	11.9 ± 5.6 aA	7.2 ± 3.0 aA	12.7 ± 2.8 aB
SF	9.8 ± 1.3 aA	14.3 ± 3.5 aB	7.9 ± 3.3 aA	7.0 ± 2.9 aA	12.5 ± 3.5 aB
HA	6.0 ± 1.2 aA	10.6 ± 2.3 aB	12.1 ± 3.8 aB	6.9 ± 3.6 aB	11.9 ± 1.8 aB
HB	6.8 ± 1.0 aA	34.2 ± 2.3 bC	11.2 ± 4.3 aB	8.8 ± 3.0 aA	13.0 ± 3.3 aB
F_25_	12.94*				
Control	12.0 ± 2.4 aA	14.7 ± 7.5 aA	14.2 ± 2.4 aA	23.0 ± 0.8 aB	11.9 ± 2.8 aA
SB	20.5 ± 2.5 bB	11.1 ± 2.2 aA	9.8 ± 2.3 aA	16.1 ± 1.2 aA	14.5 ± 1.4 aA
SR	17.6 ± 2.0 bA	10.3 ± 2.2 aA	11.8 ± 1.8 aA	20.6 ± 1.6 aB	14.3 ± 1.7 aA
SF	9.6 ± 2.8 aA	11.1 ± 2.8 aB	11.2 ± 1.7 aB	19.5 ± 1.6 aB	13.3 ± 2.7 aB
HA	11.8 ± 2.5 aA	10.4 ± 1.0 aA	11.2 ± 2.2 aA	26.0 ± 4.3 aB	15.6 ± 1.4 aB
HB	13.7 ± 3.7 aB	30.8 ± 5.5 bB	15.8 ± 1.6 aA	30.0 ± 5.1 aB	13.2 ± 4.6 aA
F_25_	12.25*				

## Discussion

The EPNs *S. brazilense*, *S. rarum, S. feltiae*, *H. amazonensis* and *H. bacteriophora* reduced hatch, number of galls, egg mass, nematodes total number and reproduction factor of *M. enterolobii*. However, only the species *S. brazilense*, *S. rarum* and *H.bacteriophora* affected juvenile survival, providing higher mortality on the 2nd day (*S. brazilense* and *S. rarum*) and on the 4th day (*H. bacteriophora*) evaluation.

The ovicidal activity of the EPNs tested can be attributed to the presence of the symbiotic bacterium. The eggs of *M. enterolobii* in the suspension can be served as a stimulus for the EPNs to release their bacteria. These bacteria may have produced substances toxic to the nematode eggs, reducing juvenile hatching (Grewal et al., 1999; Hu et al., 1999; de Freitas Ferreira et al., 2011), since the eggs of plants parasitic nematode are permeable (Perry et al., 1992).

The suspension, containing EPNs inoculated on tomato seedlings, provided a reduction in the parasitism of *M. enterolobii*, reflecting the reduction of gall number, egg mass, nematodes total number, and reproduction factor. Grewal et al. (1999) and Hu et al. (1999) observed that the toxic metabolites produced by bacteria act on the ability of *Meloidogyne’s* J2 to locate the roots. Such metabolites may have influenced the parasitism of *M. enterolobii* in tomato roots, causing a smaller number of nematodes in the root system, consequently, reducing the number of galls and egg masses.

The toxic effects of the metabolites produced by the symbiotic bacteria do not act constant in the suspension. In the course of time, occurs bacteria decline in the environment, as they do not have survival forms and, consequently, decline of the released molecules (Burnell and Stock, 2000), reducing the toxicity to juveniles of *M. enterolobii*. However, even with increasing mortality after 96 hr, it did not differ from the control, since, probably, occurred the natural mortality of juveniles that were exposed at 25°C in a suspension with accelerated metabolism.

The protein molecules produced by the bacterium *Photorhabdus* spp. (Ruiz Machado et al., 2018) present in the *H. amazonensis* and *H. bacteriphora* nematodes have been reported as presenting toxicity to *Aphelenchoides rhytium* (Massey), *Bursaphelenchus* spp., *Caenorhabditis elegans*, *Bursaphelenchus xylophilus* (Steiner and Buhrer, 1934, Kohno et al. 2007), due to the production of the secondary metabolite, 3,5-dihydroxy-4-isopropylstilbene that has nematicidal properties (Grewal et al., 1999). The other nematodes *S. feltiae, S. rarum* and *S. brazilense* have a symbiotic relationship with the bacteria of the *Xenorhabdus* genus. Bode (2009) found that *Xenorhabdus nematophila* and *Xenorhabdus bovienii* produce ketones, amides and also more complex compounds like xenocoumarins (antibiotics) that can provide toxic effect to various organisms.

Monteiro et al. (2014) reported that castor bean pie has a nematicidal effect on *Aphelenchoides besseyi* (Christie, 1942), since among the pie compounds, the ketone group is predominant. However, only *S. feltiae* has an association with the bacterium *X. bovienii* (Ehlers et al., 1997), the other EPNs studied have symbiosis with other species of bacteria of the same genus. However, it is generally believed that all bacteria of the *Steinernema* genus produce near or similar metabolites, since bacteria species of the *Xenorhabdus* genus are able of producing a wide range of bioactive compounds, including antimicrobial, antiparasitic, cytotoxic and insecticidal compounds (Fukruksa et al., 2017). Studies are needed to relate the compounds produced by the bacteria present in *S. brazilense* and *S. rarum.*

Therefore, it was verified that the reduction in hatching of *M. enterolobii* and the reduction of gall index, egg mass index, nematodes total number and reproductive factor of *M. enterolobii* in the presence *S. brazilense, S. rarum, S. feltiae, H. amazonensis*, and *H. bacteriophora*. The results demonstrate the potential of the use of EPNs in integrated management programs of *M. enterolobii.*
